# Postpartum Magnesium Sulfate Overdose: A Multidisciplinary and Interprofessional Simulation Scenario

**DOI:** 10.7759/cureus.2446

**Published:** 2018-04-07

**Authors:** Adam Garber, Purnima M Rao, Chandrew Rajakumar, George A Dumitrascu, Genevieve Rousseau, Glenn D Posner

**Affiliations:** 1 Department of Obstetrics & Gynecology, The Ottawa Hospital / University of Ottawa; 2 Department of Anesthesiology and Pain Medicine, The Ottawa Hospital / University of Ottawa; 3 Department of Obstetrics & Gynecology, University of Calgary; 4 Department of Innovation in Medical Education, University of Ottawa

**Keywords:** postgraduate medical education, simulation scenario, obstetrics and gynecology, anesthesiology, nursing, crisis resource management, adverse drug reactions, preeclampsia

## Abstract

This case is one of an eight-case multidisciplinary curriculum designed and implemented at the University of Ottawa by simulation educators with specialty training in obstetrics and gynecology (OB/GYN) and anesthesiology. Consultation with a nurse educator maintained the quality and relevance of objectives for nursing participants.

The curriculum was prepared to train OB/GYN and anesthesiology residents and labor and delivery nurses to hone crisis resource management skills and to recognize and manage rare/critical medical events in an obstetrical setting. Obstetricians, anesthesiologists, and nurses often work together in acute, high-stakes situations and this curriculum provides a safe environment to practice team-based management of such emergencies.

Over an eight-year period, this curriculum has been executed in scenario couplets in a four-year cycle to allow OB/GYN and anesthesiology residents exposure to all scenarios during a five-year residency, beginning in their second year. Prospective evaluative data has been positive. For example, over 90% of participants rated these simulations to be 5 out of 5 for “Was an effective use of my educational time” and “Will influence/enhance my future practice.”

In this scenario, participants must evaluate and treat a postpartum preeclamptic woman who is being treated with magnesium sulfate for the purpose of seizure prophylaxis. The patient experiences magnesium sulfate toxicity and subsequent respiratory arrest. Any mannequin that can display vital signs can be used for this scenario.

This simulation case includes a case template, critical actions checklist, debriefing guide, summary of key medical content, and an evaluation form for learners to provide feedback.

## Introduction

Magnesium sulfate is a commonly used medication in the labor and delivery unit. It is used for the prevention of seizures in pregnant women with preeclampsia. Magnesium sulfate toxicity may occur in up to one percent of these cases [1]. Magnesium sulfate toxicity can lead to respiratory depression or arrest. Myocardial depression and arrhythmia can also occur [1]. Maternal deaths, though rare, have been reported [2]. Management requires care from an interdisciplinary team that can provide acute medical management along with airway management. This simulation was created in order to allow anesthesiology residents, obstetrics residents, and nurses to practice the diagnosis and management of a patient with magnesium sulfate toxicity.

This scenario is part of a four-year interdisciplinary curriculum for obstetrics/gynecology (OB/GYN) and anesthesiology residents developed at the University of Ottawa Skills and Simulation Centre (see Table 1). Additional scenarios in this series can be accessed as well [3-4]. Obstetricians and anesthesiologists often work together in acute, high-stakes situations. This curriculum was designed in order to allow residents in both fields to practice both technical and crisis resource management skills in a safe and risk-free environment. Further, the interdisciplinary aspect of this curriculum allows both fields to learn about each other's knowledge, roles, and priorities during a crisis.

**Table 1:** OB/GYN Anesthesiology Nursing Simulation Curriculum MgSO4: magnesium sulfate; OB/GYN: obstetrics/gynecology

YEAR	TOPIC 1	TOPIC 2
1	Autonomic Dysreflexia	Twin Breech Delivery
2	MgSO4 Toxicity	Difficult Airway, Emergent Delivery
3	Thyroid Storm	Amniotic Fluid Embolism
4	Cord Prolapse with Abnormal Fetal Heart Rate	Postpartum Hemorrhage

The target audience is OB/GYN residents, anesthesiology residents, and practicing nurses as part of a comprehensive interdisciplinary curriculum of theatre-based simulation. Three to five learners participate in each session, and the duration of training is one hour.

The goals of the scenario are for the team to recognize and appropriately manage magnesium sulfate toxicity in a postpartum patient and to demonstrate effective crisis resource management skills in a rare and acute clinical situation. The team must work collaboratively to diagnose, resuscitate, and manage the patient appropriately.

Team objectives

(Adapted from the Ottawa Global Rating Scale [5])

1.     Demonstrate leadership skills by remaining calm and in control, making firm decisions, and maintaining a global perspective.

2.     Demonstrate problem-solving skills by identifying the diagnosis of magnesium sulfate toxicity and collaborating to provide urgent medical management while considering the most likely alternatives.

3.     Demonstrate situational awareness skills by avoiding a fixation error, reassessing and re-evaluating situations, and anticipating likely events.

4.     Demonstrate resource utilization skills by using resources to maximal effectiveness, setting clear task priority, and asking for help early.

5.     Demonstrate communication skills by communicating clearly and concisely, encouraging input and listening to staff feedback, and using directed verbal/non-verbal communication. 

OB/GYN resident objectives

1.      Demonstrate an approach to a postpartum preeclamptic patient with a decreased level of consciousness, including the consideration of a differential diagnosis.

2.     Demonstrate the management of magnesium sulfate overdose, including the administration of calcium gluconate. 

Anesthesia residents objectives

1.     Demonstrate knowledge of the critical care management of eclampsia.

2.    Demonstrate knowledge of magnesium sulfate effects in a patient with a hypertensive disorder of pregnancy.

Nursing objectives

1.     Recognize a patient with a decreased level of consciousness and call for help.

2.     Participate in the resuscitation of a patient with a magnesium sulfate overdose.

## Technical report

Case summary

A 32-year-old gravida 2 para 1 (G2P1 ) aborta 1 had a vaginal delivery 10 hours ago. She underwent induction of labor for preeclampsia at 37 weeks' gestation and delivered a singleton male infant. She is still on magnesium sulfate infusion for seizure prophylaxis. The patient developed acute renal failure due to preeclampsia and has developed magnesium sulfate toxicity. It is a busy unit, and her nurse has been pulled into another room to help out. The scenario begins when the patient’s partner/support person – played by a confederate - notices that the patient is less responsive and calls for help. The nurse responds and urgently calls the OB/GYN resident and attending physician. The patient’s oxygen saturation is low and the patient is hypotensive and tachycardic. As the patient is assessed, she becomes apneic and experiences complete respiratory arrest. The OB/GYN calls the anesthesiologist for assistance. The anesthesiologist must maintain the airway, ventilate the patient, and provide hemodynamic support.

The team, including the obstetrical nurse, obstetrical resident, and attending physician, and the anesthesiologist must consider the possible causes for this presentation, recognize magnesium sulfate overdose, and resuscitate the patient appropriately. The magnesium sulfate should be discontinued and calcium gluconate should be administered.

Learner preparation

We advocate for a comprehensive pre-briefing prior to any theatre-based simulation session, as described by Rudolph et al. [6].

Nurse: You are working on a busy labor and delivery unit.

The following information is given to the nurse independent of the other learners just prior to the start of the case: Your patient is a 32-year-old G2P1 who delivered 10 hours ago at 37 weeks' gestation after being induced for preeclampsia. She is taking labetalol 200 mg po q8h (po: per os, q8h: every eight hours). There was 1000 mL (milliliters) estimated blood loss at the time of delivery. She is also on a magnesium sulfate infusion at 1 g/h (one gram per hour) that was started during her labor. You have stepped out of the room to attend to another patient. Please respond when called in.

Junior Obstetric Resident: You are the obstetric resident on call in a tertiary hospital. Your staff obstetrician is also in the hospital.

Senior Obstetric Resident: You are the obstetrician on call in a tertiary hospital. You have an obstetrical resident also on call with you.

Anesthesiology Resident: You are the anesthesiologist on call for obstetrics in a tertiary hospital.

Setup

Equipment/Environment

This scenario takes place in a delivery room during a call shift in a tertiary care hospital.

Room: The room is to be set up as a labor and delivery suite. The room is dark with the lights turned off or low.

Mannequin: The patient is post-partum, so any mannequin with vital sign monitoring can be used. There is no need for a fetal monitor. A magnesium sulfate infusion is running by peripheral intravenous (IV) into the patient. A Foley catheter is in the bladder, draining minimal concentrated urine.

Please refer to Appendix A for a detailed list of all the required equipment.

Personnel

Simulation instructor

Simulation technician

Confederate – patient partner/supporter

Confederate – voice of mannequin

Learners

Senior anesthesiology resident – attending anesthesiologist

Senior OB/GYN resident – attending obstetrician

Junior OB/GYN resident – obstetric resident

Obstetric nurse – obstetric nurse

The baseline state of the mannequin is described in Table 2.

**Table 2:** Initial Parameters BPM: beats per minute; HPI: history of present illness; PO: per os; Q8H: every eight hours; HEENT: head, eyes, ears, nose, throat; GU: genitourinary

Initial Presentation			
Initial vital signs	Blood Pressure 110/70; Heart Rate 88 BPM; Respiratory Rate: Seven breaths per minute; SaO2 89%; Temperature: 36.5 degrees Celsius		
Overall appearance	The patient (mannequin) is lying flat on her back. Blood pressure monitor, saturation probe, and Foley catheter have been applied and the monitor is next to the patient. An intravenous (IV) bag labeled magnesium sulfate is infusing for the purpose of seizure prophylaxis. The patient’s partner/supporter is by her side, looking anxious. The patient is minimally responsive.		
Actors and roles in the room at case start	Patient – high fidelity mannequin - voiced by simulation actor who is watching the simulation from the control room. Partner/supporter – confederate actor – can be played by any willing member, including a junior resident or nurse.		
HPI	The nurse is privately pre-briefed prior to scenario start (see Learner Preparation). The patient’s partner/supporter will state that he/she has found the patient less responsive. When asked, the patient’s husband/sister will state that the patient is 32 year' old, delivered vaginally 10 hours ago, it was her second pregnancy, and she is otherwise healthy except for the preeclampsia for which the patient had her labor induced.		
Past Medical/Surgical History	Medications	Allergies	Family History
Preeclampsia in this pregnancy. Oral labetalol started one week ago for hypertension.	Prenatal vitamins, Labetalol 200 mg PO Q8H	None	None
Physical Examination			
General	Patient is very drowsy and minimally responsive with vague groans		
HEENT	Normal		
Neck	Normal		
Lungs	Decreased breath sounds due to minimal effort		
Cardiovascular	Normal		
Abdomen	Postpartum		
Neurological	Absent deep tendon reflexes – MAY NEED PROMPTING for this		
Skin	Normal		
GU	Foley to urometer with minimal concentrated urine in bag		
Psychiatric	Minimally responsive		

Ideal scenario flow

The nurse enters the room at the call of the patient’s partner/supporter to find the patient unresponsive and groaning. The room is dark or dimly lit. The patient’s partner/supporter is anxious and explains that they just recently noticed the patient to be this way. The nurse assesses the patient and then calls the obstetric resident in to assess the patient. The nurse may call the anesthesiologist as well. The nurse provides a brief handover to the resident who assesses the patient and calls the staff obstetrician and the anesthesiologist if they have not been called already. Again, handover is provided. The room lights are turned on. The patient is groaning and cannot respond to questions. The respiratory rate is seven and oxygen saturation is 89%. The medical team works through the ABCs of resuscitation and discusses possible diagnoses that might explain this presentation. They will request blood work. After recognizing the infusion of magnesium sulfate, the medical team will discontinue this infusion and administer one ampule of calcium gluconate to stabilize the myocardium. During this process of management, the patient will become apneic and the anesthesiologist will need to maintain the airway and ventilate the patient. The scenario ends once the airway is managed, the magnesium sulfate toxicity is recognized, the patient’s disposition and ongoing monitoring needs are addressed, and the patient’s partner/supporter is counseled.

Table 3 provides notes for the instructor based on branching points during the scenario based on participant actions.

**Table 3:** Flow of the Scenario bpm: beats per minute; MgSO4: magnesium sulfate; OB/GYN: obstetrician/gynecologist.

Instructor Notes – Changes and Case Branch Points		
Intervention/Time point	Change in Case	Additional Information
0-5 Minutes – initial assessment by nurse, calling for help, and handover	Obstetrical nurse in to assess patient	Patient’s partner/supporter states: “She won’t wake up!” Patient groaning
If the nurse does not call the OB/GYN or anesthesiologist		The confederate partner/supporter will say “Please get help for her”
5-10 Minutes – assessment by OB/GYN resident and attending, diagnosis, and initial management		Patient groaning; Possible MgSO4 discontinuation; Possible calcium gluconate given
7-10 Minutes – patient becomes apneic	Resp. rate decreases to zero; Oxygen Saturation decreases to 80% over two minutes; Blood pressure decreases to 90/60; Heart Rate increases to 110 bpm.	Patient stops groaning
8 minutes		Phone call from the lab providing critical results of elevated creatinine and elevated magnesium on blood work that was drawn following delivery one hour prior to the patient’s deterioration.
If the team does not recognize the respiratory arrest by the time the saturation reaches 80%		The confederate family member will say “Her lips are blue. Is she okay? What’s happening?”
If the patient is bag-mask ventilated or intubated and ventilated	Saturation improves over one minute to 96%	
If phenylephrine is given	Blood pressure will increase to 100/68 Heart rate will decrease to 98 bpm	
If ephedrine is given	Blood pressure will increase to 100/68 Heart rate will increase to 115 bpm	
10-15 Minutes – ongoing ventilatory, hemodynamic, and specific management		Possible intubation; Possible MgSO4 discontinuation; Possible calcium gluconate given
If the team calls to request more help (ICU, respiratory therapist, anesthesia assistant, second anesthesiologist)		They will be told that help will not be available for 10 minutes
15 Minutes	Team discussion about post-event monitoring; Counseling patient’s supporter	Scenario ends

Critical actions

The nurse will assess the patient and recognize a postpartum patient with a decreased level of consciousness.

The nurse will call the obstetric resident to assess the patient.

The obstetric resident will assess the patient and call the staff obstetrician and anesthesiologist.

Handover will be performed between team members as they arrive.

The room lights will be turned on.

The team will consider a differential diagnosis for the patient’s decreased level of consciousness.

The team will initiate the management of the patient with a decreased level of consciousness, decreased oxygen saturation, and hemodynamic instability.

The team will request blood work to assess blood count, kidney and liver function, and electrolytes, including magnesium levels.

The team will focus their management on a magnesium sulfate overdose, including discontinuing the magnesium sulfate and administering calcium gluconate.

Anticipated management errors

The following is a list of management errors or difficulties that are commonly encountered when using this simulation case.

Failure or Delay in Recognizing Magnesium Sulfate Toxicity

Some learners may demonstrate a difficulty in recognizing the diagnosis. Prompting may be required.  

Failure to Administer Calcium Gluconate

A more common mistake is the failure to administer calcium gluconate or the failure to administer the correct dose of calcium gluconate.

Failure to Consider Other Causes

Learners may become quickly fixated on the likely magnesium sulfate overdose and may not consider other potential causes of decreased levels of consciousness for a preeclamptic, postpartum patient.

Lack of Awareness of the Anxious Partner

Learners may have trouble managing the acute medical crisis while also managing the patient’s anxious partner.

Assessment and debriefing guide

Assessment

This scenario was developed as a formative assessment tool with a focus on nontechnical skills and crisis resource management. There are multiple suitable assessment methods that can be used. We utilize a performance checklist, as well as a crisis resource management checklist (Ottawa GRS) [5]. Please refer to Appendix B for the performance checklist.

Debriefing

We suggest an interprofessional, multidisciplinary team debriefing guided by an experienced simulation instructor. The goal of the debriefing is to allow learners to actively reflect on their own and the team’s performance, which is an essential step in adult experiential learning. Instructors should strive to create a safe, supportive, and respectful environment, where all learners are encouraged to participate. Debriefing should focus on the educational objectives, both technical and non-technical. We advocate the use of the promoting excellence and reflective learning in simulation (PEARLS) framework to organize the debriefing [7]. Where available, we advocate the use of video review during the debriefing. Be cognizant of timing; the debriefing should take 30-40 minutes. Strategies to debrief common errors can be found in Table 4, and a complete debriefing guide can be found in Appendix C.

**Table 4:** Common Errors and Debriefing Strategies SBAR: Situation-Background-Assessment-Recommendation.

Error Type	Common Errors Observed	Solutions (Teaching Points)
Technical Skills (Medical Knowledge, Clinical Skills)	Does not recognize or late to recognize respiratory depression/arrest	Allow learner to explore and reflect on possible reasons for delay and the potential impact of delay. Opportunity for advocacy inquiry technique.
	Does not recognize or late to recognize magnesium sulfate overdose	Directive feedback or group collaborative effort to develop a differential diagnosis for postpartum decreased level of consciousness.
	Does not discontinue magnesium sulfate infusion and/or does not provide IV calcium gluconate	Allow learners to reflect on possible reasons for not stopping magnesium sulfate. directive feedback regarding purpose, dose, and route of calcium gluconate administration.
Non-Technical Skills (Crisis Resource Management)	Situational awareness error – eg. manages the scenario in the dark without the room lights on.	Allow learners to reflect on strategies to maintain situational awareness.
	Resource allocation error – eg. manages the medical crisis without attending to the patient’s partner/supporter	Allow learners to reflect on the optimal way to allocate human resources.
	Communication error – eg. newly entering team members not aware of the suspicion of magnesium sulfate toxicity	Use of video playback to discuss communication techniques used or not used. Description of handover tools, such as SBAR. Discuss sharing one’s mental model or cognitive frame with specific examples.
	Fixation error – eg. team does not consider alternate causes of the decreased level of consciousness	Discuss strategies to avoid fixation error.

Program evaluation

We emphasize collecting evaluative data from participants after their simulation sessions. Our evaluation tool can be found in Appendix D. The results of our initial program evaluation can be found in Table 5, where the numbers in each column represent the number of participants who chose each number on the Likert scale. As program evaluation, this survey was exempt from Institutional Review Board approval.

**Table 5:** Evaluation Data (n = 26 Participants) CanMEDS: Canadian Medical Education Directives for Specialists

	1 (strongly disagree)	2	3	4	5 (strongly agree)
The objectives were made clear			2	3	21
The scenarios were relevant to my practice			2	4	20
The simulation team behaved in an appropriate and believable manner during the scenario			3	2	21
There was sufficient time allotted for hands-on participation and group interaction			2	4	20
The staff met the stated learning objectives			2	2	22
The staff were knowledgeable and informed			0	5	21
The staff provided adequate and appropriate feedback			2	4	20
The debriefing sessions were logically organized and clarified important issues			1	4	21
The knowledge gained from this session will enhance/influence my practice			0	3	23
The session helped increase my confidence in treating patients when a crisis occurs			3	4	19
I would like to attend additional simulation sessions				2	24
Of the 26 participants, the following identified these CanMEDS 2005 roles as having been addressed during the session					
Medical Expert			21		
Collaborator			8		
Professional			9		
Health Advocate			4		
Communicator			26		
Manager			2		
Scholar			0		
Please take a moment to reflect on your previous experience in both simulation and clinical practice. Do you think that simulation has helped your clinical practice?					
Yes			26		
No			0		

## Discussion

This scenario, as well as the rest of the OB/GYN anesthesia nursing simulation curriculum’ (Table 1), was developed to allow learners to practice managing rare and important clinical scenarios that they may not experience sufficiently during their period of training to achieve competence. Through simulation-based education, this is also an opportunity for the team to demonstrate and apply their knowledge.

Magnesium sulfate toxicity is a rare presentation. Late diagnosis and incorrect management can lead to significant morbidity and mortality for the mother and, if antepartum, the fetus as well. This scenario served to clarify both the dosing and the rationale for calcium gluconate administration for our learners, which we found to be a knowledge gap. Furthermore, the interdisciplinary practice of crisis resource management elements provides skills that can be applied to a multitude of clinical situations. The scenario begins in a darkened, quiet labor and delivery suite. Interestingly, our OB/GYN learners frequently attempted to manage this acute scenario without turning the room lights on. The anesthesia learners were much faster to recognize the darkened room as a limiting factor for acute patient care. This scenario highlighted the importance of open communication between teams and strategies to maintain situational awareness.

This scenario proved easy to run with few logistical challenges. Any mannequin with a vital sign display can be used to run the scenario. The patient’s partner/supporter can be instructed to be more or less anxious or disruptive. This can be used to modulate the cognitive load, emotional stress, and burden on available human resources. Overall, participants rated this scenario highly and felt that this scenario would improve their clinical practice.

There are limitations to this modality of teaching. A simulation requires many resources, including equipment, instructor and learner time, and instructor expertise. It is also heavily dependent on learner engagement and their willingness to participate and share their experiences with their colleagues. With the appropriate support from departments and learners, along with adequate preparation, we feel that it is an invaluable resource, especially for teaching around rare medical conditions and crisis resource management skills.

## Conclusions

We describe the design and implementation of an interprofessional and multidisciplinary simulation scenario to teach OB/GYN residents, anesthesiology residents, and nurses about the diagnosis and management of magnesium sulfate toxicity in a postpartum patient with preeclampsia. Debriefing points and common pitfalls are provided along with program evaluation data. This scenario is one component of a comprehensive theatre-based simulation curriculum, the goal of which is to provide formative assessment around less common obstetrical emergencies and crisis resource management.

## Appendices

Appendix A – equipment list

General: This scenario takes place in a delivery room during a call shift in a tertiary care hospital.

Simulation room set up as a labor and delivery room. The room is dark with the lights turned off or low. The patient's partner could be given a mannequin baby to hold during the scenario.

Mannequin

·       Mannequin on a stretcher

·       MgSO4 mini bag running through an infusion pump at 1 g/hr

·       Patient chart with a delivery note

·       Foley with minimal concentrated urine

Monitors

·       Blood pressure cuff at the bedside

·       Oxygen saturation probe on or available

Other Available Equipment

·       Oxygen mask

·       Ambubag

·       Reflex hammer

Crash cart and/or airway cart available outside the room or covered in the room:

Airway:

·  Laryngoscope

·  Oral airway

·  Endotracheal tube (#6, #7)

·  Stylet

·   Bougie

·  Laryngeal Mask Airway (#4, #5)

·  Glidescope

Breathing:

·  Stethoscope

Circulation:

·  1 L bags of crystalloid x 2

·   Intravenous (IV) infusion line x 2

Drugs:

·  Calcium gluconate

·  Epinephrine, atropine, calcium gluconate, phenylephrine, ephedrine, naloxone, labetalol, hydralazine, nifedipine, magnesium sulfate

·  Various size syringes

Appendix B – performance checklist

Anesthesiology

Performs a focused history and physical examination eliciting relevant information regarding:

o   Change in level of consciousness

o   Complications related to pregnancy and delivery

o   Past medical history

o   Current medications

Initiates resuscitation of mother:

o   Ensures IV access, Canadian Anesthesia Society (CAS) monitoring, supplemental oxygen

o   Supports airway and breathing with bag-mask ventilation and/or intubation

o   Supports circulation with supportive measures, such as IV fluids and vasopressors

o   Recognizes likely magnesium sulfate toxicity

Initiates treatment for magnesium sulfate toxicity:

o   Discontinues magnesium sulfate infusion

o   Administers calcium gluconate

Arranges appropriate disposition for the patient, for example, intensive care unit or high dependency unit

Obstetrics

·       Performs a focused history and physical examination of the patient

·       Formulates a differential diagnosis for a postpartum patient with a decreased level of consciousness

·       Initiates resuscitation of the patient

·       Recognizes likely magnesium sulfate toxicity

·       Initiates management for magnesium sulfate toxicity

o  Discontinues magnesium sulfate infusion

o   Administers IV calcium gluconate

·       Consults anesthesiology for airway management and assistance with medical management:

Nurse:

·       Completes patient assessment

·       Calls for obstetrical resident

·       Attends to patient and patient’s partner/supporter

·       Provides assistance in managing an acute medical crisis

Appendix C – debriefing guide

The following is adapted from the PEARLS debriefing guide [7]

Pre-briefing

Prior to the session, the instructor should conduct an orientation session covering the following:

Confidentiality: Instructors should sign a confidentiality form agreeing not to discuss the learners’ performance during the scenario or discussion during the debriefing outside of the simulation environment. Learners should sign a confidentiality form agreeing not to discuss any aspects of the case or debriefing outside of the simulation environment.

Equipment: Instructors should describe the function of the simulation mannequin should be discussed (what types of procedures can be performed, where to feel for pulses, where to listen for breath sounds, etc), how to operate the monitors in the room, how to get extra resources (location of phone, phone number to call, resuscitation cart, difficult airway cart, etc).

Fidelity: The learners should be encouraged to treat the scenario as they would a real clinical experience. Learners should be advised that if they are unsure that a clinical finding or event is intended or an artifact, to state this aloud so they can be appropriately directed.

Debriefing

Reactions Phase: Allows learners to express their initial thoughts and feelings

Tips:

- Ensure all learners are able to express themselves.

- Make note of any positive or negative emotions, major conflicts, or tensions that can serve as a point of discussion during the ‘Analysis’ phase.

 - Ask open-ended questions:

“How are you feeling?”

Descriptive Phase: Ensures all participants understand the major medical issues and key events of the case.

Tips:

- Ask one or two participants to summarize the case in two-three sentences to avoid a detailed play-by-play of the scenario.

- Ensure participants from all disciplines are able to contribute.

- Make note of any disagreements between the learners as to the medical content (differential diagnosis, management plan, etc), which can serve as a point of discussion during the ‘Analysis’ phase.

- Clarify any misconceptions and correct any errors regarding the main presentation and diagnosis:

“This case was meant to portray a patient with magnesium sulfate toxicity who experiences respiratory arrest.”

Key clinical points:

- Magnesium sulfate toxicity can occur in patients treated for preeclampsia. Renal dysfunction increases this likelihood.

- Magnesium sulfate toxicity can lead to respiratory depression and, ultimately, apnea, requiring urgent ventilation.

- The management of magnesium sulfate toxicity includes:

Discontinuing magnesium sulfate infusion:

- Administering one ampoule of calcium gluconate IV to stabilize the myocardium and prevent arrhythmia/myocardial depression

- Possible intubation and ventilation of the patient

- Hemodynamic support

- Considering other medical interventions including furosemide or dialysis, if appropriate.

Analysis Phase: Allows learners to practice reflective learning by exploring and analyzing positive performances and performance gaps.

Tips:

- Be genuinely curious!

- Maintain the basic assumption that everyone is smart and is doing their best for the patient!

- Focus on two to three points, including both technical and non-technical skills, in your discussion.

- Include discussion points based on your own observation as well as those generated by the learners in the "Reactions" and "Descriptive" phases.

- Select appropriate tools, including advocacy-inquiry, plus/delta, and directive feedback according to the type of performance gaps observed, the time available, the experience and insight of the learners, and your experience as a debriefer.

“What went well? What would you have wanted to change or do differently?”

“What are some pros and cons of…. (observed action or behavior)?”

“What was going through your mind when you did/said…?”

“What was your differential diagnosis?”

- Normalize mistakes.

“We specifically put you into a challenging situation and we did not expect you to manage everything perfectly.”

“In our normal practice, we are used to those around us acting in a specific way, things are different in the simulator.”

- Generalize to clinical practice.

“Have you ever experienced anything similar in your practice?”

“What strategies have you seen people use in your clinical experience?”

- Before moving to the "Summary" phase, ask the learners if there are any other issues they would like to discuss.

Summary Phase: Allows learners to review and summarize what was learned and to apply this to their clinical practice, and allows instructors to confirm if the learning objectives of the scenario were achieved.

Tips:

- Avoid bringing up new discussion points or topics.

- Ask the learners to summarize their main take-home points, i.e., “Tell me one thing you did well, one thing you would do differently, and one thing you learned that you will apply to your clinical practice.”

- Summarize the main points of the discussion.

- Ensure everyone contributes, even learners who participated as confederates can share what they learned from participating in the scenario and debriefing.

Appendix D: Evaluation form

Multidisciplinary Simulation

Date: _____________________________  Discipline: ____________________________        

Evaluation of the Session:

1 = strongly disagree, 2 = disagree, 3 = neutral, 4 = agree and 5 = strongly agree

1) The objectives of the session were explicitly stated                             1           2           3            4           5           N/A

2) The objectives were met by the simulation staff                                   1           2           3            4           5           N/A

3) Staff were knowledgeable and informed                                               1           2           3            4           5           N/A

4) There was sufficient time dedicated to hands-on                                 1           2           3            4           5           N/A

     practice

5) During the debriefing, the staff provided appropriate                           1           2           3            4           5           N/A

    and timely feedback

6) The debriefing session was organized and helped                             1           2           3            4           5           N/A

    clarify important issues with the case

7) The knowledge gained from this session will influence                      1           2           3            4           5           N/A

     my practice

8) I have gained increased confidence in my capacities                         1           2           3            4           5           N/A

    to provide care to patients in acute emergencies

9) I would enjoy having more opportunities for practice                         1           2           3            4           5           N/A

Evaluation of the Debriefers:

1 = strongly disagree, 2 = disagree, 3 = neutral, 4 = agree and 5 = strongly agree

Name of Debriefers: ________________________________________                                                 

1) The debriefers provided a safe environment to share and              1           2           3            4           5           N/A

   discuss

2) The debriefers demonstrated professionalism throughout             1           2           3            4           5           N/A

     the debriefing

3) The debriefers were engaging and facilitated my learning             1           2           3            4           5           N/A

Evaluation of the Scenario:

1 = strongly disagree, 2 = disagree, 3 = neutral, 4 = agree and 5 = strongly agree

1) The scenario was relevant to my practice                                                 1           2           3            4           5           N/A

2) The clinical encounter had high realism                                                   1           2           3            4           5           N/A

3) The degree of difficulty of the scenario was appropriate                        1           2           3            4           5           N/A

    for my level of expertise

4) The case construct was conceptually sound                                            1           2           3            4           5           N/A

    (i.e., it made sense…)

If you have answered ≤ 2 to any of the previous statements, please briefly justify:                                                                                

Global Evaluation of the experience:

1 = strongly disagree, 2 = disagree, 3 = neutral, 4 = agree and 5 = strongly agree

1) This was an effective use of my time                                                     1           2           3            4           5           N/A

2) I would appreciate having more simulation sessions                          1           2           3            4           5           N/A

3) I have gained knowledge through this experience                             1           2           3            4           5           N/A

4) What was the most useful or important lesson you learned during this session?

________________________________________________________________________________________                                                                                                                                                                               

5) Can you identify any component of this session that would need to be improved in order to make the learning experience even better?

6) Please indicate which CanMeds roles were addressed during today’s session (check all that apply)

☐ Medical Expert

☐ Collaborator

☐ Professional

☐ Health Advocate

☐ Communicator

☐ Manager

☐ Scholar
